# Geometry Selection in Three-Mirror Freeform Imagers with an Accessible Exit Pupil

**DOI:** 10.3390/s24154816

**Published:** 2024-07-24

**Authors:** Aaron Bauer, Eric M. Schiesser, Jannick P. Rolland

**Affiliations:** The Institute of Optics, University of Rochester, 480 Intercampus Drive, Rochester, NY 14627, USA

**Keywords:** freeform, three mirror anastigmat, reimaging

## Abstract

Reimaging telescopes have an accessible exit pupil that facilitates stray light mitigation and matching to auxiliary optical systems. Freeform surfaces present the opportunity for unobscured reflective systems to be folded into geometries that are otherwise impracticable with conventional surface types. It is critical, however, to understand the limitations of the enabled folding geometries and choose the one that best balances the optical performance and mechanical requirements. Here, we used the aberration theory of freeform surfaces to determine the aberration correction potential for using freeform surfaces in reimaging three-mirror telescopes and established a hierarchy for the different folding geometries without using optimization. We found that when using freeform optics, the ideal folding geometry had 9× better wavefront performance compared to the next best geometry. Within that ideal geometry, the system using freeform optics had 39% better wavefront performance compared to a system using off-axis asphere surfaces, thus quantifying one of the advantages of freeform optics in this design space.

## 1. Introduction

During the first stages of an optical design, it is critical to understand the physical boundaries into which the optical system must be packaged. In the case of an unobscured reflective system, the physical boundaries not only dictate the overall dimensions of the system but also the geometries into which it can be folded. Within the various folding geometries (FGs), rotationally variant aberrations are present that require correction for a clear image to be resolved. The most basic surface shapes, spheres, offer little benefit for rotationally variant aberration correction, except for in specific configurations [1,2]. Similarly, centered aspheres cannot impact the rotationally variant aberrations prevalent in asymmetric packages. Thus, for decades, designers have utilized off-axis sections of otherwise rotationally symmetric conics and aspheres to manage the aberrations of unobscured reflective systems [3,4,5]. However, limiting the surfaces to off-axis sections of rotationally symmetric parent surfaces was primarily conducted to facilitate the manufacture of such surfaces at the time, but they were not the ideal surface shapes for aberration correction. As manufacturing techniques such as diamond machining [6,7,8], ion beam figuring [9,10,11], and CNC grinding and polishing [12,13,14,15,16,17] matured, it became feasible to make surfaces without an axis of symmetry within or beyond the aperture, termed freeform surfaces [18]. By utilizing every surface shape degree of freedom during design, it was seen that improvements to critical optical parameters such as system volume [19,20], optical performance [19,20,21,22,23,24], zoom ratio [25,26], and throughput could be achieved.

While the ability of freeform surfaces to take any shape seems like the ultimate optical design tool, they still operate under the same physical rules as conventional surfaces—they must simultaneously correct all the aberrations across a continuous object field-of-view (FOV). Fuerschbach et al. developed a mathematical framework that predicts the aberrations that can be corrected by a specific shape of a freeform surface described by Zernike polynomials [27]. We leveraged that framework to develop a design method for using freeform surfaces that starts with choosing an FG that inherently has aberrations that are correctable using freeform surfaces [28]. It was shown that for a given set of specifications, there exist many FG options, but the majority are limited by combinations of aberrations that simply cannot be corrected with freeform surfaces (or any other surface shape). Thus, when given the option of various FGs, it is critical to identify the optimal solution.

To date, there has been some investigation of the optical behavior of the FGs of a non-reimaging freeform three-mirror imager (also known as the reflective triplet) [28,29], but to our knowledge, the FGs of reimaging freeform three-mirror imagers have yet to be explored. Here, the aberrations of the various FGs for a reimaging freeform three-mirror imager, where the presence of an intermediate image and accessible exit pupil impact the aberrations inherent to an FG, will be studied, along with how freeform optics can be used to correct these aberrations. This type of system is often referred to as a three-mirror anastigmat (TMA) [5]. The reimaging property of a TMA is useful when stringent stray light control is required or when pupil-matching to an auxiliary optical system. In the process, we will identify the hierarchy of FGs for freeform TMAs by optimizing a 250 mm aperture system operating at F/3 with a 2° × 2° full FOV in the visible spectrum. For the top-tier FG, we will perform an additional study of the tradeoffs with system volume. Lastly, a direct comparison between a freeform design and an equivalently specified off-axis asphere design will be provided to quantify the freeform advantage in this space.

## 2. Folding Geometry Investigation

The choice of which specific FG to use varies on a case-by-case basis. In systems where the optical system drives the packaging, the FG with the best performance can be used. However, in cases where the optical system plays a supporting role, there is often a prescribed area carved out for the optics, with little freedom for choosing the FG. In that case, it is important to understand when a particular FG can be used and when it should be avoided. In this section, we will show how to predict the freeform correction potential of an FG without performing a full-system optimization.

### 2.1. Introduction to Folding Geometries

**Figure 1:**
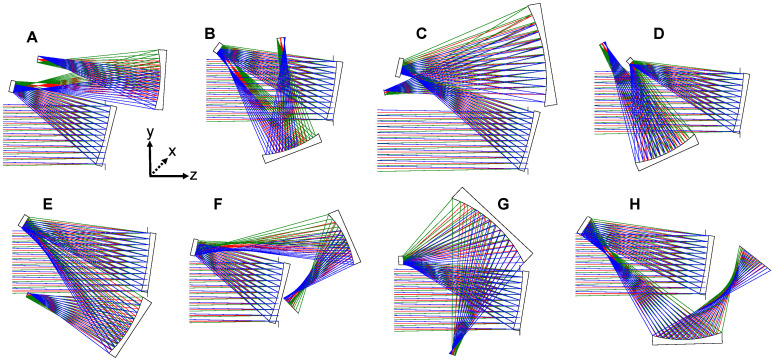
Folding geometries for a three-mirror imager with reimaging. Each different folding geometry (**A**–**H**), is considered distinct because the secondary or tertiary mirror is tilted in a new direction or the image plane crosses over the incoming ray bundles. These systems have only spherical surfaces and are not yet optimized for image quality. These systems operate at F/3 with a 250 mm entrance pupil and a 2° × 2° full FOV. Each ray bundle of a certain color corresponds to a specific point in the FOV.

In the context of an unobscured three-mirror imager, an FG refers to one of the various permutations of mirror tilt directions combined with the image plane location. With Y-Z planar symmetry assumed (see coordinate axes in Figure 1), to clear the outgoing rays after reflection from any given mirror, that mirror can either be tilted clockwise or counterclockwise about its local *X*-axis. For a system with three mirrors that can each rotate in two directions, there are (2) × (2) × (2) = 8 possible mirror tilt permutations. However, due to the Y-Z planar symmetry of these systems, a clockwise or counterclockwise tilt of the primary mirror yields optically equivalent systems, so the number of mirror tilt permutations is reduced to four. Within each tilt permutation, the image plane location adds sub-options (e.g., crossing or not crossing various ray bundles with the image plane, like in Figure 1d or Figure 1e) that define the possible FGs. For the specifications detailed in Table 1, we arrive at a total of eight different FGs, as illustrated in Figure 1.

Tilting the mirrors to create an unobscured system generates significant aberrations that must be dealt with, to obtain a quality image. The direction and magnitude of each mirror’s tilt determine the orientation and magnitude of the resulting aberrations; thus, each FG has a different combination of tilt-induced aberrations [30]. Fuerschbach et al. [27] detail the combinations of aberrations that are correctable using freeform surfaces, so we look for those in each FG. Specifically, there are four main low-order aberrations that are orders of magnitude larger than the others at the geometry selection stage of the design. They are as follows: field-constant astigmatism (FCA), field-asymmetric field-linear astigmatism (FAFLA), field-constant coma (FCC), and field-linear medial field curvature, also called focal plane tilt (FPT). If these four main low-order aberrations are not corrected efficiently, a high-performance system is unlikely to be found.

### 2.2. Design Study Parameters

As noted, a TMA is a three-mirror imager that has an intermediate image and an accessible exit pupil. To satisfy both requirements, the aperture stop location is preferably placed at the primary mirror (or in object space), as we want to keep the pupil plane separated from the intermediate image plane. The airspaces between mirrors are unsuitable for the aperture stop due to the lack of space to place a physical aperture without obscuring another part of the system. From Fuerschbach et al. [27], we can understand the effect of putting a freeform surface on each of the three mirrors. With the primary mirror serving as the aperture stop, freeform shapes at this surface can only correct field-constant aberrations. The secondary mirror’s close proximity to the intermediate image means that it will provide a highly field-dependent freeform correction, with only a small field-constant contribution. The footprints of each field on the secondary mirror are small, requiring a large freeform shape contribution to yield any significant aberration response. Finally, the tertiary mirror will be located roughly at the mid-point between the relayed pupil and the intermediate image, so it will have field-dependent and field-constant aberration responses that are similar in magnitude. The full specifications for the design are shown in Table 1.

### 2.3. Using Geometry Filters

In Figure 1, we show the eight unique FGs into which this system could be packaged. One could optimize each FG with freeform surfaces to find which FGs are optimal, but that is a time-consuming task and becomes exponentially more tedious as more surfaces are added to the system. Instead, as we conducted for the reflective triplet design [28], we construct filters to apply to each FG to ascertain its potential for freeform correction without needing a full optimization. The filters are based on the aberration theory of freeform surfaces documented in [27] and focus on the four main low-order aberrations described in Section 2.1.

**Figure 2:**
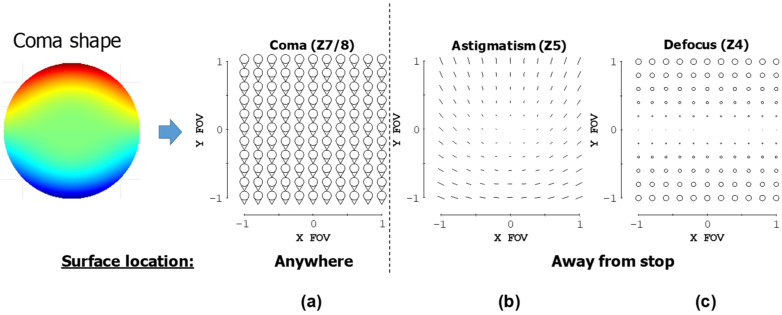
The resulting aberrations from adding a freeform coma shape to a surface in the optical system are shown here in aberration full-field displays, as predicted by the aberration theory of freeform surfaces [27]. When the coma surface is located at the aperture stop, only (**a**) field-constant coma is produced. When the coma surface is moved away from the aperture stop, (**a**) field-constant coma, (**b**) field-asymmetric, field-linear astigmatism, and (**c**) focal plane tilt are produced.

FCA is of minor concern, as it can be corrected by putting an astigmatism-shaped surface on any surface in the system, irrespective of the FG. Ideally, it would be placed at the stop surface (primary mirror) to minimize the distortion that it also induces when placed away from the stop. Thus, the first filter addresses the correction of the FAFLA and FCC inherent in each FG. As illustrated in Figure 2 and from [27], we know that a coma-shaped freeform surface simultaneously adds contributions of FAFLA and FCC when located away from the aperture stop. The relative orientation between the FAFLA and FCC contributions depends on if the surface is placed before or after the aperture stop (planar symmetry allows for only two relative orientations between FCC and FAFLA). In our case, there are two surfaces that follow the aperture stop; thus, only a single relative orientation between the FAFLA and FCC can be contributed from a coma surface, and therefore, only that relative orientation is correctable using the freeform surfaces. Filter #1 checks the FAFLA and FCC inherent in each FG to identify if the correct relative orientation is present and, thus, if it is correctable using freeform surfaces. If it is, a single coma surface can correct both aberrations simultaneously and efficiently.

The second filter addresses the third aberration that a coma surface generates, the FPT. When correcting the FAFLA and FCC, as described in the previous filter, some amount of FPT is also added. Filter #2 checks to see if the added FPT serves to decrease the FPT that is inherent to the FG. If it does, the single coma surface has the potential to correct the FAFLA, FCC, and FPT contributions simultaneously. It is important to correct the FPT optically rather than by using a tilted detector to avoid image distortion and the reduced responsivity of the detector [31].

The final filter uses more conventional optical design wisdom and is applied more subjectively than the previous two. Extreme surface tilts used to clear the rays generate more low-order and higher-order aberrations than can be corrected using freeform surfaces. Additionally, with the constraint of an accessible exit pupil, if the distance between the tertiary mirror and the closest feasible spot for the exit pupil (i.e., the location nearest to the tertiary mirror, where it is clear of all overlapped ray bundles) is too great, then the first-order optics limit the possible aberration correction. Filter #3 checks the FGs for those two aspects.

### 2.4. Starting Geometry Construction

Before the filters can be applied to the FGs, a starting-point optical model for each FG must be created. As emphasized in our earlier work [28], an all-spherical starting point is sufficient, and accordingly, it is not critical for the starting designs to have good optical performance. On the contrary, it is fully expected that the starting points will be significantly aberrated. The first-order constraints that the starting points must satisfy are the system focal length, the Petzval correction, and for the system to be unobscured. A basic procedure for setting up these three-mirror starting points is as follows: the airspaces between the primary/secondary mirrors and secondary/tertiary mirrors should be approximately equal and chosen to fill the allowable system length (which is assumed to be a known parameter). The focal length of the primary mirror should be 1.25×–1.5× larger than primary/secondary airspace so that the intermediate image forms between the secondary and tertiary mirrors. Then, the powers of the secondary and tertiary mirrors can be determined by enforcing the system focal length and zero Petzval curvature constraints. Finally, the mirrors should be tilted the minimum amount that results in complete unobscuration. The fine-tuning of the mirror powers can be performed manually if there are obscuration challenges for any given FG. Again, the actual mirror powers and airspaces are not critical in these starting points.

### 2.5. Applying the Filters to the FGs

By evaluating the FGs using these three aberration-based filters, each FG can be put into a tier based on how many and which filters are satisfied, where a greater number of satisfied filters predicts better aberration correction. We made starting designs for and applied the filters to the FGs shown in Figure 1, with the results summarized in Table 2. Included in the table is whether the FG has the ability to have an accessible intermediate image, at which a field stop can be placed to further limit stray light.

As an example of applying Filter #3, which is the most subjective filter, let us consider Geometry G. After reflection from the tertiary mirror, the light must cross two bundles of rays—those going into the tertiary mirror and those entering the system towards the primary mirror—before forming an image, which is a long optical path relative to some of the other FGs. Thus, with a fixed system F-number, the tertiary mirror is going to be quite fast. Further, the tertiary mirror must be tilted substantially to move the image plane clear of the rays entering the system. The combination of a fast mirror being substantially tilted results in prohibitive levels of aberration. Additionally, the exit pupil must also be made accessible, thus tightly constraining the distance between it and the tertiary mirror and dictating the first-order surface properties instead of those properties being driven by aberration correction.

Based on the results of the filtering, there is one Tier 1 FG that unequivocally has the best potential for correction using freeform surfaces. This FG is the preferred FG for conventional TMAs and has been shown to be optimal for freeform reflective triplet designs as well [28]. The lower tier designs should not be deemed as unusable in a global sense, but rather their suboptimal performance should be of consideration when balancing mechanical and optical requirements. Further differentiation between the Tier 2 designs is challenging as higher-order aberrations play a larger role, and the applied filters focus solely on the low-order aberrations.

**Figure 3:**
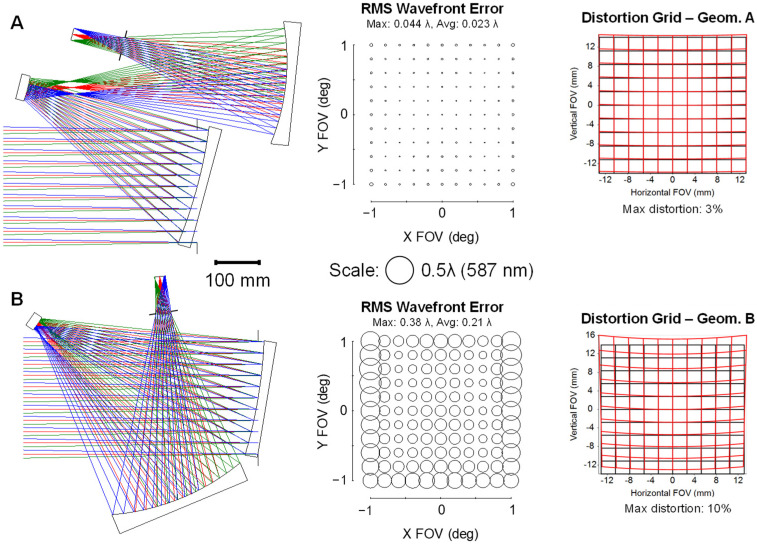
Optimized designs using freeform surfaces for Tier 1 Geometry (**A**) and Tier 2 Geometry (**B**). These were the only two FGs to achieve <0.25 waves’ average RMS WFE at a volume of 70 L. The RMS WFE of Geometry A is ~9× better than Geometry B. Distortion grids for both designs indicate that Geometry A has approximately 3× lower distortion.

To complete this exercise and validate the filtering results, we optimized all eight FGs with a target bounding box volume constraint of 70 L with clearance constraints such that there is adequate spacing between the optics for mechanics/electronics. The aberration-based method outlined in [28] was followed for each optimization. Given that the design method we followed was based on using Zernike polynomial freeform surfaces, we also employed Zernike polynomial freeform surfaces here (up to Z28 in Fringe ordering). Geometries A and B were the only FGs whose performance at 70 L was <0.25 waves root-mean-squared (RMS) averaged over the FOV. However, Geometry A performed 9x better in RMS wavefront error (WFE) and had 3× lower distortion than Geometry B, demonstrating the superiority of that form. Distortion in this case is measured as the distance between the real chief ray and the paraxial chief ray for each field point at the image plane. Distortion was constrained in the design process by controlling the (x,y) intersection points of the real chief rays at the image plane to meet the distortion goals. The optimized designs for Geometries A and B are shown in Figure 3.

Geometry A was independently found to be the ideal geometry for reimaging freeform systems based on a survey of the three-mirror imager landscape leveraging confocal conics, though at a slower speed (F/5) and smaller entrance pupil (500 mm) [32]. Geometry A was also leveraged in a diffraction-limited F/1.7, 4° × 0.1° FOV, 90 mm entrance pupil design operating in the LWIR [33], showing that the geometry is flexible for different applications and specifications.

The quality of the exit pupil in the designs optimized in this work was not constrained, though the final designs do have reasonably well-formed pupils due to their limited FOVs. For the case where an aperture would be put at the exit pupil plane to mitigate stray light, an ill-formed exit pupil would mostly result in some vignetting of the off-axis fields. If an application calls for more strict control on the quality of the exit pupil and/or more FOV is added, additional design constraints can be enforced [34].

## 3. Volume Study of Tier 1 FG

**Figure 4:**
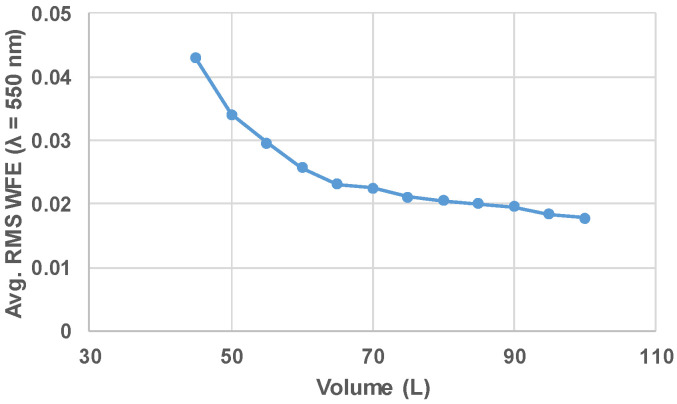
The average RMS WFE is plotted vs. the system volume for the Tier 1 FG (Geometry A). Volumes below 45 L were unable to be optimized to have diffraction-limited performance over the full FOV.

Given the overwhelming benefit of using the Tier 1 FG over the other FGs, it is instructive to extend the design study to include how the Tier 1 FG performs over a range of volumes. With the performance goal of diffraction limited at 550 nm over the full FOV, the most compact package size achievable was 45 L. To make the system even more compact, the mirror powers must increase. Additionally, the tilts necessary to unobscure the system become greater as the system becomes more compact. These two factors contribute to an increase in the aberrations of the system before adding freeform surfaces, especially higher-order aberrations, which are more difficult to correct without also impacting the low-order terms. The average RMS WFE values across the field vs. volume for the resulting designs are shown in Figure 4. The plot illustrates that there is a quick end to volume reduction while still maintaining diffraction-limited performance over the full field.

Reimaging three-mirror imagers have less design freedom than their non-reimaging counterparts given the additional constraints/benefits of having an accessible intermediate image and exit pupil. Using the roadmap in Bauer et al. [35], it can be seen that a non-reimaging freeform three-mirror imager, designed with the same specifications as shown in Table 1, has a volume that is approximately 70% of the smallest diffraction-limited reimaging freeform three-mirror imager. For additional comparisons between non-reimaging and reimaging systems at other specifications, a separate roadmap for reimaging systems is needed.

## 4. Comparison to Off-Axis Aspheres

**Figure 5:**
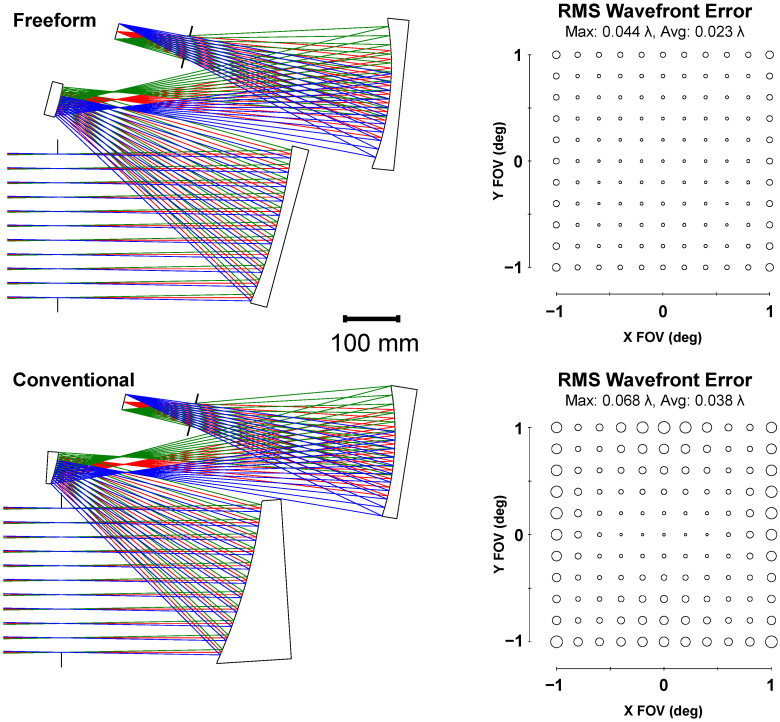
A comparison of the (**top**) freeform solution to the (**bottom**) conventional solution using off-axis aspheres. Each design fits within a 70 L volume. The freeform design has a 39% better average RMS WFE and a 35% better maximum RMS WFE compared to the conventional design with equivalent specifications.

Often, when approaching an optical design, there is a tendency to stick to what is known or commonly conducted. However, when the benefit of a new technology is substantial, the risk of trying something new is outweighed. To speed up the adoption of freeform optics, it is necessary to perform direct comparisons to their conventional counterparts to quantify the improvements they offer. Here, we optimized a conventional TMA using off-axis aspheres with equivalent specifications to the 70 L freeform TMA described above in Table 1. Both the freeform and conventional designs together with their respective RMS WFE analyses are shown in Figure 5. In this scenario, freeform surfaces enable a performance improvement of 39% in the average RMS WFE and 35% in the maximum RMS WFE. The conventional design shown in Figure 5 is right at the diffraction limit of 0.07 waves max across the FOV. As indicated in Section 3, the most compact freeform design that maintained diffraction-limited performance across the full FOV was 45 L, representing a 36% decrease in volume compared to the conventional design with the same performance.

## 5. Conclusions

By analyzing the aberrations of the various FGs for a freeform TMA, one can make an informed choice about how to best integrate an optical system into an overall system design. Here, we applied three filters to the FGs based on the aberration correction abilities of freeform surfaces, following a procedure documented in the literature. This allowed us to create a hierarchy of FGs and conserve time that would otherwise be spent optimizing each individual FG (though we also included an example of the latter to validate the predictions). The Tier 1 FG showed a 9× better RMS WFE compared to the next best FG, underscoring the point that freeform surfaces are not an aberration silver bullet for all systems and that, for TMA systems, designers should stick to the Tier 1 FG when image quality is a top (or even medium) priority. The Tier 1 FG is the same geometry that has been used for years in conventional off-axis TMA designs, and through this study, we now have an aberration-based understanding of the why it works so well. Finally, we demonstrated the freeform advantage in TMAs by comparing them to a conventional design using off-axis aspheres, showing a significant performance gain.

It should be noted that the increased performance must often be weighed against the increased manufacturability challenges. Freeform optics is an emerging technology, where the departures from symmetries can make the processes of fabrication and metrology more complex and, therefore, more costly. Additionally, the folded geometries of freeform systems may require intricate optomechanical designs so that the optics can be properly located and mounted in three dimensions while meeting alignment tolerances. In the designs presented here, the overall layout of the conventional design and the freeform design are quite similar, including the speed of the base surfaces, so we do not expect that the freeform system will be any more challenging to assemble. However, in general, by using concurrent engineering best practices when designing freeform systems, the manufacturing complexities, costs, and sensitivities can be minimized.

## Figures and Tables

**Table 1:** Design specifications for the freeform TMA study.

Parameter	Specification
Entrance pupil diameter [mm]	250
F/#	3
Full field-of-view [deg]	2 × 2
Root-mean-squared wavefront error [waves]	<0.07 (diffraction limited)
Analysis wavelength [nm]	550
Distortion [%]	<5
Volume [L]	Various

**Table 2:** The results of the filtering of the FGs. “Yes” means the FG passed the filter, and N/A indicates that it was not necessary to test the filter due to failing Filter #3. Each FG was placed into a tier, where a lower numbered tier indicated better performance was to be expected. Access to an intermediate image plane is also noted.

Geometry	Filter #1	Filter #2	Filter #3	Tier	Int. Img. Access
A	Yes	Yes	Yes	1	Yes
B	No	No	Yes	2	No
C	Yes	No	Yes	2	Yes
D	Yes	No	Yes	2	No
E	Yes	No	Yes	2	No
F	N/A	N/A	No	3	Yes
G	N/A	N/A	No	3	Yes
H	N/A	N/A	No	3	No

## Data Availability

Data is available from the authors upon reasonable request.
